# Effect of different head position during tracheal intubation on postoperative sore throat: a randomized clinical trial

**DOI:** 10.1080/07853890.2025.2464943

**Published:** 2025-02-14

**Authors:** Tao Shan, Huimin Zhang, Xiao Zhou, Hongguang Bao, Liu Han, Chuan Su, Qilian Tan, Jun Yin, Tao Dan

**Affiliations:** ^a^ Department of Anesthesiology, Perioperative and Pain Medicine, Nanjing First Hospital, Nanjing Medical University, Nanjing, China; ^b^ Wuxi Taihu University, Wuxi, China; ^c^ Center for Global Health, Department of Pathogen Biology and Immunology, Jiangsu Key Laboratory of Pathogen Biology, State Key Lab of Reproductive Medicine, Nanjing Medical University, Nanjing, China

**Keywords:** Airway management, intubation, position, postoperative complications, sore throat

## Abstract

**Introduction:**

Postoperative sore throat is the most frequently complaint after tracheal intubation. We aimed to determine whether changing patients’ head position during intubation reduces the incidence of postoperative sore throat.

**Methods:**

We randomized 130 patients receiving oral tracheal intubation into one of the two groups: the sniffing position group and elevation position group. Patients in the sniffing position group maintained sniffing position consistently during intubation, while those in the elevation position group transitioned from the sniffing position to the elevation position during tube advancement to the trachea. The primary outcome was incidence of airway trauma and postoperative sore throat (none/mild/moderate/severe) 1 h after surgery. The secondary outcomes were the incidence of postoperative sore throat at 6 h,12 h and 24 h, and hoarseness at 1 h, 6 h,12 h and 24 h postoperatively.

**Results:**

One hundred twenty-eight patients completed our trial. There were no differences in the baseline characteristics of the patients between the sniffing position and elevation position group [51 (14.8) vs 53 (15.5) for age, 25/39 vs 26/38 for sex (male/female)]. No difference in basic airway condition was observed. Transitioning patient’s head from sniffing to elevation position during tube advancement to tracheal resulted in a significantly lower incidence of airway trauma [10/64 vs 23/64, risk ratio (95% CI): 0.76 (0.61–0.94), *p* = 0.009], postoperative sore throat and hoarseness compared with maintaining the sniffing position at 1 h [10/64 vs 30/64, risk ratio (95% CI): 0.63 (0.49–0.81), *p* < 0.001 for sore throat; 22/64 vs 34/64, risk ratio (95% CI): 0.71 (0.52–0.98), *p* = 0.044 for hoarseness] and 6 h [4/64 vs 17/64, risk ratio (95% CI): 0.78 (0.67–0.92), *p* = 0.006 for sore throat; 12/64 vs 27/64, risk ratio (95% CI): 0.71 (0.56–0.91), *p* = 0.002 for hoarseness]. There were no significant differences in postoperative sore throat and hoarseness at 12 and 24 h between the two groups.

**Conclusions:**

Transitioning patients’ head position from the sniffing position to a head elevation position during tube advancement into tracheal could significantly reduce the incidence of airway trauma, postoperative sore throat and hoarseness.

**Trial registration number:**

ChiCTR2300073198

## Introduction

Postoperative sore throat (POST) and hoarseness are frequent and distressing complications associated with tracheal intubation under general anaesthesia, contributing to increased postoperative morbidity and patient dissatisfaction [1]. These complications rank as the eighth most common complication, with reported incidence rates ranging from 12.1% to 100% within the initial 24 h postoperatively [2–4]. Specifically, a multicenter prospective cohort study found that POST occurred in 61.8% of patients with endotracheal intubation [5]. The impact of POST on patient outcomes is significant, as it can lead to increased healthcare costs due to extended hospital stays and additional treatments [6]. Furthermore, POST can negatively affect patients’ quality of life, causing discomfort and reducing patient satisfaction [7].

The tracheal mucosa releases inflammatory mediators after intubation, indicating that the aetiology of POST is likely an inflammatory process caused by mucosal injury of the trachea or vocal cords [8,9]. Common preventive measures to reduce the occurrence of POST include the application of lubricating jelly over the tracheal tube and the use of anti-inflammatory drugs [3]. While lubricating jelly or anti-inflammatory drugs might provide some relief, they are limited in their ability to address the root cause of POST. Strategies should focus on reducing mechanical trauma and providing long-term protection to the laryngeal mucosa, ultimately improving patient outcomes and quality of life. Superior laryngeal nerve block has been employed as a method to prevent POST by blocking efferent stimulation, but local mucosal or vocal cord injury did not alleviate [10]. Choosing a smaller tube size, thermal softening of the tracheal tube and reducing extraction force during stylet removal appeared to attenuate airway trauma during intubation, thereby reducing the incidence of POST [11,12]. Hence, alleviation of vocal cord or tracheal mucosal injury is crucial for reducing both POST and hoarseness.

Sniffing position and head elevation position are recommended for intubation when using a video laryngoscope [13,14]. Study indicated that the head elevation position significantly reduced intubation difficulty and the need for manoeuvres to advance the tube through the glottis, compared to sniffing position. Mucosal or vocal cord injuries may vary during tube advancement based on different head and neck positions. In clinical, we noticed easier advancement of the endotracheal tube into the tracheal with less resistance, when transitioning the patient’s head from the sniffing position to the elevation position. In the sniffing position, the angle between the tracheal tube and trachea increased while intubation. Elevating the head and neck during intubation can improve the alignment of the airway axis, potentially mitigating the mechanical pressure on the laryngeal mucosa, minimizing the risk of injury. However, the potential influence of this transition of head and neck position during intubation on airway injury and POST remains unknown.

Therefore, we hypothesized that when transitioning patient’s head from the sniffing position to the elevation position during intubation may alleviate airway injury. This prospective, randomized study was conducted to investigate the effect of changing head position during endotracheal tube advancement on the airway injury and incidence of POST in patients receiving general anaesthesia. The findings of our study on the impact of head position during intubation on POST and airway trauma may have significant clinical implications that could inform changes of head positions in standard intubation practices.

## Materials and methods

### Ethics

This prospective, single-centre, randomized study was approved by the Institutional Ethics Board of Nanjing First Hospital with Jianping Gu being the president of the ethics committee in Nanjing, China, on 30 March 2023 (No. KY20230330-05). The study was registered in the Chinese Clinical Trial Registry (https://www.chictr.org.cn, ChiCTR2300073198, Date of registration: 4 July 2023). Written informed consent was obtained from all subjects before trial commencement. Our trial adheres to the applicable CONSORT guidelines and principles stated in the Declaration of Helsinki.

### Patients and randomization

We allocated patients aged 18–65 years, classified as ASA physical status I-II, scheduled for abdominal or lower extremity surgery and undergoing oral tracheal intubation under general anaesthesia. The exclusion criteria were as follows: (1) pre-existing sore throat or hoarseness before anaesthesia; (2) cervical spine disease; (3) upper respiratory infection or a history of tracheostomy; (4) Mallampati score ≥ 3; and (5) participation in other clinical trials within the past 3 months. We used a computer-generated random number table to allocate the patients into two groups in a 1:1 ratio using SPSS 19.0 (IBM, Chicago, IL, USA), depending on the head position during intubation. Patients remained unaware of their group assignments throughout the study. An anaesthesiologist who was not involved in the study prepared sealed opaque envelopes containing group assignments.

### Intervention

All patients received 8-hour fasting period before operation. Upon arrival in the operating room, the patients were monitored using electrocardiography, noninvasive blood pressure and pulse oximetry. A pillow of 7 cm height was placed under the patient’s occiput. Without premedication, general anaesthesia was initiated with midazolam at a dosage of 0.03 mg/kg, propofol at 1.5 mg/kg, sufentanil at 0.5 μg/kg and rocuronium at 0.6 mg/kg. Before tracheal intubation, an otorhinolaryngologist, who was blinded to the group allocation, examined the airway mucosa using a fibreoptic bronchoscope (TIC-SD-III, UE Medical Co, Ltd, China) with external diameters 4.2 mm and took the pictures for later comparison.

**Figure 1.**
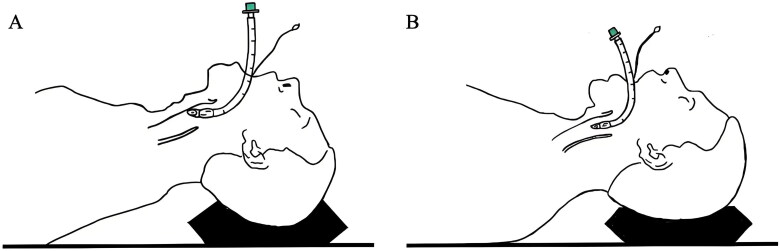
Association of endotracheal tube and airway infront wall during intubation between different head positions. (A) Sniffing position (B) Elevation position.

Single-use tracheal tubes (Covidien, USA) featuring low-pressure, –high-volume cuffs were employed for routine intubation in our centre. Tubes with internal diameters of 7.5 and 7.0 mm were utilized for male and female patients, respectively. Oral intubation was performed using a video laryngoscope (TD-C-IV, UE Medical Co, Ltd, China) by an experienced anaesthesiologist, with all patients maintaining a sniffing position to fully expose the glottis. The tracheal tube, preloaded with a stylet, was then bent to match blade’s curvature. The anterior segment of the tracheal tube was inserted into the glottis under direct visualization. In the sniffing group, after the initial insertion, the patient’s head was maintained in the sniffing position, stylet was removed by another anaesthesiologist and tracheal tube was simultaneously inserted. In the elevation group, the patients’ head positions were changed from the sniffing position to the elevation position, with the jaws moving towards the sternum after stylet removal, while tracheal intubation was completed, as depicted in Figure 1. If resistance was encountered during the tube insertion, the tube was gently rotated clockwise. The tube position was reconfirmed with direct visualization using a video laryngoscope after tube insertion and PetCO_2_ monitoring. Throughout the procedure, silicone oil was applied to the tracheal tube and stylet to reduce the resistance. A force measuring device (SH-II, Nscing Es, China) was employed to accurately measure the extraction force upon stylet removal, ensuring that the maximum extraction force remained below 10 N in all patients [12].

Immediately after intubation, the tracheal tube cuff was inflated, and cuff pressure was maintained at 25 cm H_2_O using a cuff pressure measurement device (Cuff Pressure Gauge, 109-02, Covidien, USA). All tracheal tubes were secured in the midline using medical tape. Mechanical ventilation in the volume control mode was sustained during the procedure, maintaining a tidal volume of 6–8 ml/kg, PEEP of 3–5 cm H_2_O and PetCO_2_ at 35–40 mmHg. Anaesthesia was maintained with continuous infusion of propofol at a rate of 4–6 mg/kg/h and remifentanil at a rate of 10–20 μg/kg/h. Rocuronium was administered intravenously as needed during surgery. Analgesia during surgery was supplemented with flurbiprofen axetil 100 mg intravenously. Tropisetron (4 mg) and sufentanil (10 μg) were administered intravenously 20 min before the end of surgery. Postoperatively, patient-controlled intravenous analgesia was initiated using sufentanil (150 μg) with a total volume of 250 ml, delivered at a continuous rate of 3 ml/h and a 5 ml bolus with a lockout interval of 8 min.

After surgery, the patients were transferred to the post-anaesthesia care unit. We continuously administered remifentanil to maintain patients’ tube tolerance until patients regained consciousness. When the patients regained complete consciousness and adequate spontaneous breathing and response to verbal commands, the oropharynx was carefully suctioned using a soft disposable suction catheter. Neuromuscular blockade was reversed with atropine (0.02 mg/kg) and neostigmine (0.05 mg/kg). Along with tracheal extubation, the same otorhinolaryngologist used the flexible laryngoscopy through the trachea tube and reexamined airway mucosa lesions. Photographs were taken for comparison with the initial findings prior to intubation. All patients subsequently received oxygen *via* a nasal cannula.

### Measurements of outcomes

We assessed the Mallampati score, thyromental distance, mouth opening and laryngoscopic views using the Cormack–Lehane grades before tracheal tube intubation.

The primary outcome in this trial was the incidence of sore throat 1 h after surgery and airway mucosa lesions. The lesions were categorized based on their location and nature as follows: petechiae, small red spots on the mucosa; oedema, swollen mucosa; haematoma, bleeding into the mucosa; granuloma, granulation tissue remained as localized and rounded tissue; and any other lesions [15]. Only the most prominent lesion was documented for analysis, if multiple lesions were observed.

Secondary outcomes included the incidence of sore throat at 6, 12 and 24 h postoperatively, as well as hoarseness at 1, 6, 12 and 24 h postoperatively. POST was graded as follows: none (no sore throat), mild (pain with deglutition), moderate (pain constantly present and increasing with deglutition), or severe (pain interfering with eating and requiring analgesic medication). Hoarseness was defined as an acoustic quality differing from the preoperative voice and graded as follows: none (no hoarseness), mild (noticed by the patient), moderate (obvious to the observer) and severe (aphonia) [11]. Individuals experiencing hoarseness without pain were excluded from the POST category. All outcomes were evaluated by the same anaesthesiologist blinded to group allocation of the patients.

### Statistical analysis

The sample size for this study was determined based on prior research reporting POST incidence ranging from 12.1% to 100% under general anaesthesia. Considering the specific conditions of our centre, we assumed a POST incidence of 46% in our clinical centre. Utilizing PASS 15.0 software, we calculated the sample size according to our preliminary test results. In our preliminary test, the incidence of sore throat was 22% in the elevation position group. The calculated sample size was 58 patients in each group with a power of 80% and an alpha error of 5%. Accounting for a potential 10% dropout rate, we finally randomized 130 patients in our trial.

Continuous variables were assessed for normal distribution using the Kolmogorov-Smirnov test and expressed as mean (standard deviation) or median (interquartile range). Based on the distribution analysis, the independent Student’s t-test or Mann-Whitney U-test was used to compare statistical differences in continuous variables. Categorical variables are presented as the number of patients and were compared using Pearson’s chi-square test or Fisher’s exact test. For the primary outcomes, the risk ratio with 95% confidence interval was calculated. Statistical analysis was performed using SPSS software (version 19.0; SPSS Inc., IBM, Chicago, IL, USA). All reported *p* values were two-sided and significance was set at *p* < 0.05.

## Results

**Figure 2.**
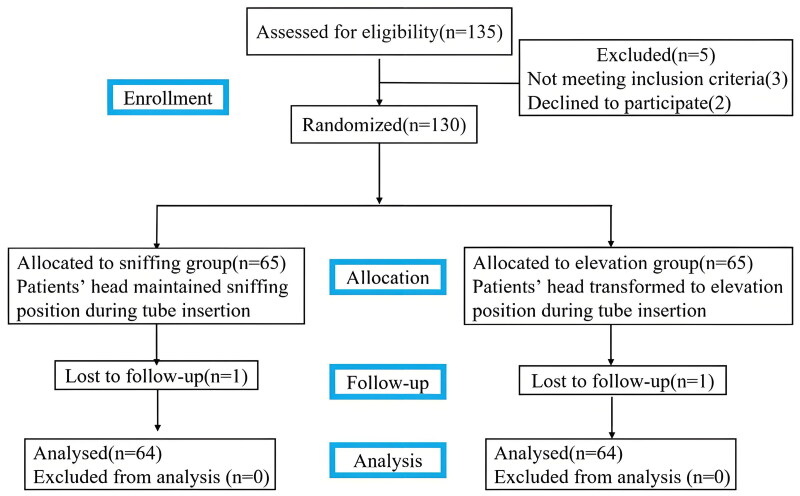
CONSORT of diagram.

We enrolled 135 eligible patients between July 2023 and December 2023, and five patients subsequently were excluded from the study. One patient in the sniffing group experienced massive bleeding and required reoperation for bleeding control, and one patient in the elevation group was transferred to the ICU for intraoperative bleeding. Ultimately, 128 patients completed the study (Figure 2). No significant differences were observed in the baseline characteristics of the patients, airway characteristics, surgical or anaesthesia duration between the two groups (Table 1).

**Table 1.** Demographic characteristics and data related to surgery. Values are mean (standard deviation).

	Sniffing group (*n* = 64)	Elevation group (*n* = 64)	*p*
Age (yr)	51 (14.8)	53 (15.5)	0.649
Sex (M/F)	25/39	26/38	0.857
BMI (kg m^-2^)	24.0 (2.9)	23.7 (3.4)	0.585
ASA(I/II)	12/52	9/55	0.474
Mallampati score (1/2)	21/43	23/41	1.000
Thyromental distance (cm)	7.4 (0.6)	7.37 (0.7)	0.892
Mouth opening (cm)	4.3 (0.5)	4.45 (0.7)	0.314
Cormack–Lehane grades(I/II/III/IV)	34/24/6/0	36/23/5/0	0.695
Intubation time(s)	15.0 (2.3)	15.3 (1.8)	0.443
Duration of anaesthesia (min)	85 (80.0)	105 (68.8)	0.301
Duration of surgery (min)	70 (83.8)	87.5 (67.5)	0.328

Data are presented as mean ± standard deviation or number. Data presented as mean ± standard deviation were compared using an independent Student’s t-test. Data presented as number of patients were compared using Pearson’s chi-square test.

**Figure 3.**
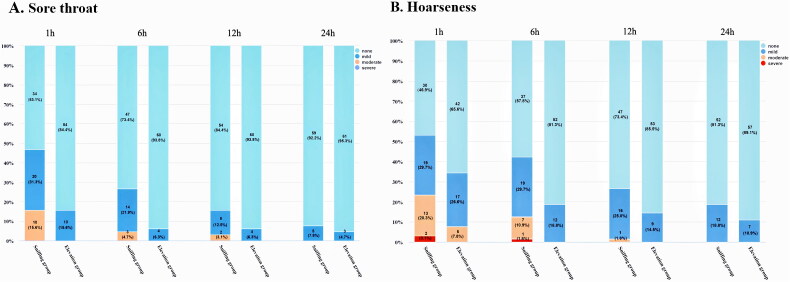
Incidence of sore throat and hoarseness in each time point between different head positions.

Incidence of airway trauma was lower in the elevation group [10/64 vs 23/64, risk ratio (95% CI): 0.76 (0.61–0.94), *p* = 0.009] (Table 2). The incidence of sore throat was markedly lower in the elevation group than the sniffing group at both 1 h [10/64 vs, 30/64, risk ratio (95% CI): 0.63 (0.49–0.81), *p* < 0.001] and 6 h [4/64 vs 17/64, risk ratio (95% CI): 0.78 (0.67–0.92), *p* = 0.006] postoperatively. The incidence of hoarseness was lower in the elevation group at both 1 h [22/64 vs 34/64, risk ratio (95% CI): 0.71 (0.52–0.98), *p* = 0.044] and 6 h [12/64 vs 27/64, risk ratio (95% CI): 0.71 (0.56–0.91), *p* = 0.002] postoperatively than in the sniffing group. There were no significant differences in POST and hoarseness at 12 and 24 h between the two groups (Table 3 and Figure 3). The highest incidences of sore throat and hoarseness were reported at 1 h after surgery, with no newly developed symptoms observed at 6, 12 and 24 h postoperatively. None of the patients experienced severe POST. In the sniffing group, two patients reported severe hoarseness, whereas in the elevation group, one patient experienced severe hoarseness (Figure 3).

**Table 2.** Incidence of airway mucosa lesions. Values are number (%) of patients.

	Sniffing group (*n* = 64)	Elevation group (*n* = 64)	Risk ratio (95% CI)	*p*
Petechiae	15	5	0.83 (0.71–0.97)	0.015
Oedema	6	4	0.97 (0.87–1.07)	0.510
Haematoma	2	1	0.98 (0.93–1.04)	1.000
Total incidence[n(%)]	23 (35.9)	10 (15.6)	0.76 (0.61–0.94)	0.009

Data are presented as number of patients. Data presented as number of patients were compared using Pearson’s chi-square test or Fisher exact test.

CI: confidence interval.

**Table 3.** Incidence and severity of POST and hoarseness between the sniffing and elevation group.

	Sniffing group (*n* = 64)	Elevation group (*n* = 64)	Risk ratio (95% CI)	*p*
Sore throat				
1 h	30	10	0.63 (0.49–0.81)	<0.001
6 h	17	4	0.78 (0.67–0.92)	0.006
12 h	10	4	0.90 (0.80–1.02)	0.181
24 h	5	3	0.97 (0.88–1.06)	0.718
Total incidence	30	10	0.63 (0.49–0.81)	<0.001
Hoarseness				
1 h	34	22	0.71 (0.52–0.98)	0.044
6 h	27	12	0.71 (0.56–0.91)	0.002
12 h	17	9	0.86 (0.72–1.03)	0.149
24 h	12	7	0.91 (0.79–1.06)	0.320
Total incidence	34	22	0.71 (0.52–0.98)	0.033

Data are presented as number of patients. Data presented as number of patients were compared using Pearson’s chi-square test or Fisher exact test.

CI: confidence interval.

## Discussion

Our trial aimed to compare the impact of two different head positions during tracheal tube advancement on the incidence of airway injury, POST and hoarseness. The results revealed a notable decrease in the incidence of airway injury, sore throat and hoarseness when the patient’s head was transformed to an elevation position, as opposed to patients maintaining the sniffing position during tube advancement into the trachea.

The occurrence of POST is influenced by various factors, including the type of airway device used, insertion technique, lubricant choice, cuff pressure, duration of surgery and patient demographics such as age and gender [16–19]. Interestingly, the expertise of the anaesthesiologist performing tracheal intubation does not significantly impact the incidence of POST in adults [20]. Numerous non-pharmacological and pharmacological methods, have been explored to mitigate POST, with varying degrees of success [21–23]. Collectively, these findings suggest that the aetiology of POST involves mucosal erosion of the tracheal tube cuff, trauma from tracheal intubation and mucosal dehydration [24,25]. Consequently, the key focus in minimizing POST lies to alleviation of tracheal injury caused by tracheal intubation.

Diverse head and neck positions can influence the advancement of the tracheal tube. A prior investigation comparing the impact of pillow height (4 vs 12 cm) on tracheal tube intubation suggested that altering the head position with a higher pillow significantly facilitated smoother progression of the tracheal tube through the glottis, resulting in a lower incidence of sore throat [26]. Kim and colleagues observed a lower incidence of laryngeal pressure or lifting force when advancing the tracheal tube into the glottis in the head elevation position than in the sniffing positions [14]. However, they did not estimate the incidence of POST. In our trial, we found reduced airway mucosa injury when transition patients’ position to elevation position. In our trial, the cumulative incidence of POST within 24 h after surgery was notably high (46.9%) in patients maintaining the sniffing position, which is consistent with previous studies [2–4]. While changing the patients’ head position to an elevation position during tube advancement resulted in a significant decrease in the airway mucosa injury and POST (15.6%).

The sniffing position plays a pivotal role in achieving alignment of the three crucial axes during orotracheal intubation and becomes a fundamental aspect of anaesthesiology training [13,27,28]. In our study, we uniformly positioned all patients in the sniffing position by employing a UE video laryngoscope with a 40° distal curvature of the blade to expose the glottis [29]. To navigate the tracheal tube ‘around the corner’, we used a rigid stylet to conform the tube to the blade curvature [30,31]. Nevertheless, extracting the stylet from the tracheal tube can be challenging because of its inherent compliance and the original curvature of the stylet, potentially altering the morphology of the tube front and causing airway trauma and oedema. The extraction of the stylet may result a phenomenon termed the ‘stylet extraction effect’. When excessive extraction force is applied to remove the stylet, the tracheal tube itself must support the additional force, extend the tip of the tracheal tube forward into the tracheal and anteriorly to impinge the inner wall of the trachea. This additional force is involuntarily applied to the larynx at an abnormal angle, causing direct mucosal damage. When the laryngeal axis is tilted upward in the sniffing position, the angle between the tracheal tube and trachea increases, making tube advancement into the trachea more challenging [32]. In line with this hypothesis, our trial implemented a technique in which we transformed the patients’ head positions from the sniffing position to an elevation position during stylet extraction and tube advancement. This adjustment aimed to reduce the angle between the front part of the trachea and laryngeal axis, potentially mitigating the impingement force originating from the tracheal tube tip and anterior wall of the trachea. As a result, in our study, this modification facilitated a smaller angle between the tube tip and the trachea, leading to a reduction in mucosal injury. Consequently, tracheal advancement became more manageable, ultimately contributing to a decrease of airway injury, POST and hoarseness.

Notably, our study identified the highest incidence of POST occurring at 1 h after surgery, deviating from previous reports of peak incidence at 2 or 6 h [10]. This discrepancy challenges the convention that POST is more likely at 2 h owing to the metabolism of anaesthetic drugs [33–35]. Nevertheless, a previous report showed that continuous intravenous opioid postoperatively did not lead to a reduction in POST^12^. Our study is consistent with this finding. Further studies are warranted to evaluate this possibility.

Our study also had several limitations. Firstly, anaesthesiologists who performed tracheal intubation were not blinded to the patients’ head positions. Secondly, our evaluation focused exclusively on adult patients age range 18–65 years. Consequently, the generalizability of our findings to elderly patients, paediatric populations, or pregnant women may be limited. Finally, we did not assess the influence of head position transition on patients with difficult airway. To validate our findings, future studies should include larger, multicenter trials to apply and expand on our results.

In summary, our trial demonstrates that transitioning patients’ head and neck position from the sniffing position to an elevation position during tracheal tube advancement efficiently reduces the incidence of airway mucosa injury, POST and hoarseness. Consequently, we may advocate the routine adoption of this simple postural transformation technique during tracheal intubation.

## Supplementary Material

CONSORT Checklist.doc

## Data Availability

All data relevant to the study are included in the article. The data supporting the findings of this study are available from the corresponding author upon reasonable request.

## References

[1] Aqil M, Khan MU, Mansoor S, et al. Incidence and severity of postoperative sore throat: a randomized comparison of Glidescope with Macintosh laryngoscope. BMC Anesthesiol. 2017;17(1):127. doi: 10.1186/s12871-017-0421-4. 28899338 PMC5596501

[2] Higgins PP, Chung F, Mezei G. Postoperative sore throat after ambulatory surgery. Br J Anaesth. 2002;88(4):582–584. doi: 10.1093/bja/88.4.582. 12066737

[3] El-Boghdadly K, Bailey CR, Wiles MD. Postoperative sore throat: a systematic review. Anaesthesia. 2016;71(6):706–717. doi: 10.1111/anae.13438. 27158989

[4] Sumathi PA, Shenoy T, Ambareesha M, et al. Controlled comparison between betamethasone gel and lidocaine jelly applied over tracheal tube to reduce postoperative sore throat, cough, and hoarseness of voice. Br J Anaesth. 2008;100(2):215–218. doi: 10.1093/bja/aem341. 18024955

[5] Bekele Z, Melese Z. Incidence and risk factors for postoperative sore throat after general anesthesia with endotracheal intubation: prospective cohort study. Ann Med Surg (Lond). 2023;85(6):2356–2361. doi: 10.1097/MS9.0000000000000786. 37363454 PMC10289720

[6] Javed H, Olanrewaju OA, Ansah Owusu F, et al. Challenges and solutions in postoperative complications: a narrative review in general surgery. Cureus. 2023;15(12):e50942. doi: 10.7759/cureus.50942. 38264378 PMC10803891

[7] Liljas AEM, Brattström F, Burström B, et al. Impact of integrated care on patient-related outcomes among older people—a systematic review. Int J Integr Care. 2019;19(3):6. doi: 10.5334/ijic.4632. PMC6659761 31367205

[8] Lee JY, Sim WS, Kim ES, et al. Incidence and risk factors of postoperative sore throat after endotracheal intubation in Korean patients. J Int Med Res. 2017;45(2):744–752. doi: 10.1177/0300060516687227. 28173712 PMC5536682

[9] Mathew G, Agha R, Group S. STROCSS 2021: strengthening the reporting of cohort, cross-sectional and case-control studies in surgery. Ann Med Surg (Lond). 2021;72:103026. doi: 10.1016/j.amsu.2021.103026. 34820121 PMC8599107

[10] Chen Z, Jin Y, Lu G, et al. Preoperative ultrasound-guided internal branch block of superior laryngeal nerve reduces postoperative sore throat caused by double lumen endotracheal intubation: a randomized trial. Anesth Analg. 2023;137(6):1270–1278. doi: 10.1213/ANE.0000000000006534. 37227947

[11] Seo JH, Cho CW, Hong DM, et al. The effects of thermal softening of double-lumen endobronchial tubes on postoperative sore throat, hoarseness and vocal cord injuries: a prospective double-blind randomized trial. Br J Anaesth. 2016;116(2):282–288. doi: 10.1093/bja/aev414. 26787799

[12] Kusunoki T, Sawai T, Komasawa N, et al. Correlation between extraction force during tracheal intubation stylet removal and postoperative sore throat. J Clin Anesth. 2016;33:37–40. doi: 10.1016/j.jclinane.2015.12.024. 27555130

[13] Akihisa Y, Hoshijima H, Maruyama K, et al. Effects of sniffing position for tracheal intubation: a meta-analysis of randomized controlled trials. Am J Emerg Med. 2015;33(11):1606–1611. doi: 10.1016/j.ajem.2015.06.049. 26227445

[14] Kim H, Chang JE, Won D, et al. Effect of head and neck positions on tracheal intubation using a McGRATH MAC video laryngoscope: a randomised, prospective study. Eur J Anaesthesiol. 2023;40(8):560–567. doi: 10.1097/EJA.0000000000001838. 37052067

[15] Knoll H, Ziegeler S, Schreiber JU, et al. Airway injuries after one-lung ventilation: a comparison between double-lumen tube and endobronchial blocker: a randomized, prospective, controlled trial. Anesthesiology. 2006;105(3):471–477. doi: 10.1097/00000542-200609000-00009. 16931978

[16] Fenta E, Teshome D, Melaku D, et al. Incidence and factors associated with postoperative sore throat for patients undergoing surgery under general anesthesia with endotracheal intubation at Debre Tabor General Hospital, North central Ethiopia: a cross-sectional study. International Journal of Surgery Open. 2020;25:1–5. doi: 10.1016/j.ijso.2020.06.003.

[17] Lee S-Y, Shih S-C, Leu Y-S, et al. Implications of age-related changes in anatomy for geriatric-focused difficult airways. Int J Gerontol. 2017;11(3):130–133. doi: 10.1016/j.ijge.2016.11.003.

[18] Zhao X, Cao X, Li Q. Dexamethasone for the prevention of postoperative sore throat: a systematic review and meta-analysis. J Clin Anesth. 2015;27(1):45–50. doi: 10.1016/j.jclinane.2014.06.014. 25468585

[19] Choi EK, Baek J, Kim DY. Effect of dexmedetomidine and remifentanil infusion on postoperative sore throat after lumbar spine surgery in the prone position. Medicine (Baltimore). 2023;102(14):e33506. doi: 10.1097/MD.0000000000033506. 37026907 PMC10082256

[20] Inoue S, Abe R, Tanaka Y, et al. Tracheal intubation by trainees does not alter the incidence or duration of postoperative sore throat and hoarseness: a teaching hospital-based propensity score analysis. Br J Anaesth. 2015;115(3):463–469. doi: 10.1093/bja/aev234. 26243647

[21] el Hakim M. Beclomethasone prevents postoperative sore throat. Acta Anaesthesiol Scand. 1993;37(3):250–252. doi: 10.1111/j.1399-6576.1993.tb03709.x. 8517099

[22] Thomas S, Beevi S. Dexamethasone reduces the severity of postoperative sore throat. Can J Anaesth. 2007;54(11):897–901. doi: 10.1007/BF03026793. 17975234

[23] Uztüre N, Menda F, Bilgen S, et al. The effect of flurbiprofen on postoperative sore throat and hoarseness after LMA-proseal insertion: A randomised, clinical trial. Turk J Anaesthesiol Reanim. 2014;42(3):123–127. doi: 10.5152/TJAR.2014.35693. 27366405 PMC4894219

[24] Combes X, Schauvliege F, Peyrouset O, et al. Intracuff pressure and tracheal morbidity: influence of filling with saline during nitrous oxide anesthesia. Anesthesiology. 2001;95(5):1120–1124. doi: 10.1097/00000542-200111000-00015. 11684980

[25] Navarro RM, Baughman VL. Lidocaine in the endotracheal tube cuff reduces postoperative sore throat. J Clin Anesth. 1997;9(5):394–397. doi: 10.1016/s0952-8180(97)00068-8. 9257206

[26] Deguchi S, Komasawa N, Kido H, et al. Impact of pillow height on double-lumen endotracheal tube intubation with McGRATH MAC: a prospective randomized clinical trial. J Clin Anesth. 2016;34:339–343. doi: 10.1016/j.jclinane.2016.05.024. 27687405

[27] Genc A, Karaman T, Karaman S, et al. The effect of head position on glottic visualization with video laryngoscope and intubation success in obese patients who are not expected to have a difficult airway: a prospective randomized clinical study. J Clin Monit Comput. 2022;36(6):1785–1793. doi: 10.1007/s10877-022-00827-z. 35141803

[28] Carassiti M, Zanzonico R, Cecchini S, et al. Force and pressure distribution using Macintosh and GlideScope laryngoscopes in normal and difficult airways: a manikin study. Br J Anaesth. 2012;108(1):146–151. doi: 10.1093/bja/aer304. 21965048

[29] Xue FS, Yang BQ, Liu YY, et al. Current evidences for the use of UEscope in airway management. Chin Med J (Engl). 2017;130(15):1867–1875. doi: 10.4103/0366-6999.211536. 28748861 PMC5547840

[30] Lee YC, Lee J, Son JD, et al. Stylet angulation of 70 degrees reduces the time to intubation with the GlideScope(R): A prospective randomised trial. J Int Med Res. 2018;46(4):1428–1438. doi: 10.1177/0300060517741065. 29332445 PMC6091841

[31] Cook TM, Woodall N, Frerk C, et al. Major complications of airway management in the UK: results of the Fourth National Audit Project of the Royal College of Anaesthetists and the Difficult Airway Society. Part 1: anaesthesia. Br J Anaesth. 2011;106(5):617–631. doi: 10.1093/bja/aer058. 21447488

[32] Levitan RM, Heitz JW, Sweeney M, et al. The complexities of tracheal intubation with direct laryngoscopy and alternative intubation devices. Ann Emerg Med. 2011;57(3):240–247. doi: 10.1016/j.annemergmed.2010.05.035. 20674088

[33] Chang JE, Kim H, Han SH, et al. Effect of endotracheal tube cuff shape on postoperative sore throat after endotracheal intubation. Anesth Analg. 2017;125(4):1240–1245. doi: 10.1213/ANE.0000000000001933. 28368938

[34] Hung NK, Wu CT, Chan SM, et al. Effect on postoperative sore throat of spraying the endotracheal tube cuff with benzydamine hydrochloride, 10% lidocaine, and 2% lidocaine. Anesth Analg. 2010;111(4):882–886. doi: 10.1213/ANE.0b013e3181d4854e. 20304980

[35] Park JJ, Huh H, Yoon SZ, et al. Two-handed jaw thrust decreases postoperative sore throat in patients undergoing double-lumen endobronchial intubation: A randomised study. Eur J Anaesthesiol. 2020;37(2):105–112. doi: 10.1097/EJA.0000000000001149. 31860598

